# Modification of topoisomerases in mammospheres derived from breast cancer cell line: clinical implications for combined treatments with tyrosine kinase inhibitors

**DOI:** 10.1186/1471-2407-14-910

**Published:** 2014-12-03

**Authors:** Refael Peleg, Marianna Romzova, Inga Kogan-Zviagin, Ron N Apte, Esther Priel

**Affiliations:** The Shraga Segal Department of Microbiology, Immunology & Genetics, Faculty of Health Sciences, Ben-Gurion University of the Negev, Beer Sheva, Israel

**Keywords:** Cancer stem cells, Topoisomerase, CPT, Etoposide, Erlotinib, Gefitinib

## Abstract

**Background:**

Accumulating evidences suggest that tumors are driven by a small population of cells, termed “cancer stem cells” (CSCs), which may be resistant to current therapeutic approaches. In breast carcinoma, the CSCs have been identified as a CD44^+^/CD24^−^ cell population. These rare cells are able to grow as non-adherent sphere-like structures, termed “mammospheres”, which enables their isolation and expansion in culture. To design efficient strategies for the complete eradication of CSCs, it is important to identify enzymes and proteins that are known as anti-cancer targets, and differ in their properties from those present in the none CSCs. Here we investigated the activity and expression of type I and type II DNA topoisomerases (topo I and topo II) in CSCs and their response to anti-topoisomerase inhibitors.

**Methods:**

MCF7 breast cancer cells, PC3 prostate cancer cells and 4 T1-Luc-Oct3/4pG mouse mammary carcinoma cells were grown on low-attachment dishes in specific medium and allowed to form spheres. Enrichment of CSC population was verified by immunostaining, flow cytometry or fluorescent microscopy imaging. Nuclear protein extracts were prepared and topoisomerases activity and protein levels were determined. Cell viability was examined by the MTT and Neutral Red assays.

**Results:**

Unlike the adherent MCF7 cell line, topo I activity is decreased and topo II activity is increased in the CSCs. However, the relative levels of the enzyme proteins were similar in both mammospheres and adherent cells. Topo I activity in mammospheres is regulated, at least in part, by PARP-1, as observed by the recovery of topo I activity after treatment with PARP-1 inhibitor 3-Aminobenzamide. Mammosphere-derived cells show reduced sensitivity to topo I inhibitor, camptothecin, and increased sensitivity to topo II inhibitor etoposide. Intact mammospheres show increased resistance to both drugs. A combined treatment of intact mammospheres with either CPT and gefitinib, or etoposide and erlotinib, increased the anti-cancer effect of both drugs.

**Conclusions:**

The data of this study suggest that the understanding of biological behavior of essential enzymes such as topoisomerases, in CSCs’ progression and early stages of tumor development, is important for developing new strategies for cancer treatment as well as new therapies for advanced disease.

**Electronic supplementary material:**

The online version of this article (doi:10.1186/1471-2407-14-910) contains supplementary material, which is available to authorized users.

## Background

Over the past few decades there has been significant progress in cancer treatment and overall survival. In addition, a decreased mortality for some of the common epithelial malignancies, such as breast and prostate cancers, is being observed, mainly due to early detection and prevention. However, the survival of patients with metastatic diseases has not been dramatically changed [1].

In the past few years, accumulating evidence suggest that most malignancies are generated by a subset of cells, termed “cancer stem cells” (CSCs), which may be responsible for tumor resistance to current therapeutic approaches [1, 2] and can give rise to new tumors after long remissions, probably due to their unique properties of longevity, quiescence, and self-renewal, similar to normal stem cells [3]. In addition to these properties, cancer stem cells express active telomerase, activated anti-apoptotic and repair pathways, and increased multi-drug resistance (MDR) transporters and migration ability, all of which play an important role in tumor development and progression [1, 4].

Evidence for the existence of CSCs were found in many types of cancer, including brain, pancreatic and head and neck cancers, in addition to those mentioned above [2], which led to the question of whether current anti-cancer treatments target the right cells [1].

For human breast cancer, the population of cells displaying CSC properties was identified and found to express the CD44^+^/CD24^–\low^ cell markers. The unique ability of these cells to grow in suspension and form sphere-like structures, termed “mammospheres”, allows their isolation and expansion *in vitro*[5, 6]. This system has become a tool for understanding the underlying mechanisms that regulate stem/progenitor cells’, or cancer stem cells’, essential activities and pathways [2].

From a clinical outlook, the CSC concept has significant therapeutic implications, as these cells need to be completely eradicated in order to provide long-term disease-free survival and prevent tumor recurrence or metastasis. The common therapeutic approach includes cytotoxic compounds that can trigger cell death mechanisms in highly proliferating cancer cells. Quiescent CSCs are thought to be more resistant to chemotherapy, as they express a slow turnover rate [4, 7]. However, most CSCs appear to evade cytotoxic therapies or irradiation through active mechanisms, such as ATP-binding cassette (ABC) transporter proteins overexpression, active DNA repair systems, and expression of anti-apoptotic factors [4, 8].

To design efficient strategies for the complete eradication of CSCs, it is important to identify enzymes and proteins that are known as anti-cancer targets, and differ in their properties from those present in the tumor cell populations, that are not designated as CSCs.

DNA topoisomerases are essential nuclear enzymes that work to resolve topological problems that normally occur during DNA metabolism. In humans, the family of DNA topoisomerases consists of 6 enzyme proteins, which are divided into two major groups – type I- and type II-topoisomerases. Their involvement in crucial DNA associated-processes, such as replication, transcription and repair, marked them as a target of several anti-cancer agents which are used today in the treatment of various malignancies [9, 10]. The catalytic activity of topoisomerases involves the formation of transient covalent bridges of enzyme-DNA complexes. At the interface of these complex, topoisomerase I interacts with camptothecin (CPT), and several of its analogs (i.e. topotecan, irinotecan), and topo II interacts with etoposide, to induce cell-death [11–13].

In previous studies we and others have demonstrated the increased anti-cancer effect of topoisomerase inhibitors in combination with other anti-cancer agents, such as tyrosine kinase inhibitors [14–17].

In this study we investigate the activity of topoisomerases in cancer stem cells (CSCs) at early stages of tumor development, and evaluate their sensitivity to topoisomerases inhibitors, alone and in combination with tyrosine kinase inhibitors. Here we show, for the first time, that topoisomerases are differentially regulated in mammospheres, compared with the parental adherent cells, and that these alterations in enzymatic activity modulate the sensitivity of cells to anti-cancer treatments.

Understanding the biological behavior of essential enzymes in CSCs, as well as the evaluation of their sensitivity to clinically used anti-cancer drugs, might lead to better, more effective treatment protocols.

## Methods

### Cells

MCF7 human breast cancer cell line was kindly received from Prof. Etta Livneh, Ben-Gurion University, Beer Sheva, Israel. 4 T1-Luc-Oct3/4pG mouse mammary cancer cell line was kindly received from Dr. Zvi Granot, Hebrew University, Jerusalem, Israel. These cells were cultured as monolayer in DMEM medium (Beit HaEmek Biological Industries, Israel). PC3-human prostate cancer cell line was kindly received from Prof. Shraga Segal, Ben-Gurion University, Beer Sheva, Israel, and cultured as monolayer in RPMI 1640 medium (Beit HaEmek Biological Industries, Israel). Media were supplemented with 10% fetal bovine serum, 100 units/ml penicillin, 100 μg/ml streptomycin, and 0.29 mg/ml L-glutamine (Beit HaEmek Biological Industries, Israel). Cell lines were grown in a humidified incubator supplemented with 5% CO_2_, at 37°C.

### In vitro isolation and expansion of stem-like cancer cells

MCF7 and 4 T1-Luc-Oct3/4pG cells were cultured as single cells on non-adherent plates, at a density of 20,000 cells\ml, in the presence or absence of fetal bovine serum, to form sphere-like structures (mammospheres). Cells grown without serum were cultured in DMEM:F12 medium solution mix, supplemented with 0.4% bovine serum albumin (BSA), 20 ng/ml EGF (Sigma-Aldrich, Israel), 10 ng/ml bFGF (Beit HaEmek Biological Industries, Israel), and 5 μg/ml insulin (Sigma-Aldrich, Israel). Mammospheres were collected after 7–10 days in culture. PC3 cells were cultured as prostaspheres in the same manner, except that a RPMI:F12 medium solution mix was used, and 20 ng/ml bFGF were added.

### Antibodies and compounds

Supercoiled DNA plasmid pUC19 was purchased from Fermentas (Hanover, MD, USA). Kinetoplast DNA was purchased from TopoGen, USA. Erlotinib (Tarceva®) was kindly provided by Roche Diagnostics GmbH Pharma Research, Penzberg, Germany. Gefitinib (Iressa™) was kindly provided by AstraZeneca Pharmaceuticals (Cheshire, UK). Stock solutions of erlotinib and camptothecin (Sigma, Israel), at 20 mM, and of gefitinib, at 50 mM, were dissolved in DMSO (Sigma-Aldrich, Israel), stored in aliquots at −80°C and diluted in DMSO before being added to the reaction mixture or to the cell culture medium. Stock solution of etoposide (Teva, Israel), at 34 mM, was stored at room temperature.

The primary antisera were as follows: mouse monoclonal anti-β-actin antibody (MP Biomedicals LLC, USA); rabbit monoclonal anti-CD44 antibody (Epitomics Inc., USA); rabbit polyclonal anti-CD133 antibody (Abcam, USA); mouse anti-γH2AX antibody (Abcam, USA); goat polyclonal anti-Lamin B (Santa Cruz Biotechnology Inc., USA); goat polyclonal (C-15) anti-topo I (Santa Cruz Biotechnology Inc., USA); goat polyclonal anti-Topo IIα (Santa Cruz Biotechnology Inc., USA); mouse monoclonal anti-CD24 antibody was kindly provided by Prof. Mina Fogel, Kaplan medical center, Israel. Appropriate horseradish peroxidase secondary antibodies were purchased from Santa Cruz Biotechnology Inc. Enhanced chemiluminescence (ECL) reagents were purchased from Biological Industries Beith Haemek, Israel. Appropriate fluorescent secondary antibodies were purchased from Jackson Immunoresearch laboratories Inc., USA. DAPI (4′,6-diamidino-2-phenylindole) was purchased from Sigma-Aldrich, Israel.

### Determination of cellular markers by Immnocytostaining

Adherent MCF7 cells or mammosphere-derived cells were seeded onto Lab-Tek™ glass chamber slides (Thermo Scientific, USA) and allowed to adhere overnight. Cells were fixed with methanol:acetone for 10 min and then washed with PBS. After blocking with BSA for 30 min, primary antibody was added and cells were stored overnight at 4°C. Cells were then washed and the corresponding secondary antibody was added for 1 h, in the dark, at 4°C. DAPI was added, after which cells were washed, chambers were detached from the slide, and cells were mounted and covered. Images were obtained by confocal microscopy.

### Determination of cellular marker by flow cytometry

Adherent MCF7 cells and mammospheres were collected and dissociated enzymatically and mechanically. Cells were washed with PBS and blocked for 30 min with 1% BSA, in PBS, on ice. Cells were then centrifuged at 1200 rpm and supernatant was discarded. Extracellular staining was performed by adding a mouse-anti CD24 antibody (courtesy of Prof. Mina Fogel, Kaplan Medical Center, Israel) to the corresponding samples and stored on ice. Then, cells were fixed with 1% formaldehyde for 10 min at room temperature. Samples were washed with 1% BSA, in PBS, and cell membrane was permeabilized by adding 0.1% Triton for 10 min, at room temperature. Intracellular staining was performed by adding rabbit anti-CD44 (Epitomics, USA) to the corresponding samples and stored on ice. Samples were washed and corresponding secondary antibodies – FITC conjugated anti-mouse IgG (Jackson Immunoresearch Laboratories, Inc.) and AlexaFluor 633-conjugated anti-rabbit IgG (Life Technologies) – were used, according to the manufacturer’s instructions and stored for 30 min on ice, in the dark. Samples were washed and resuspended in PBS, after which they were analyzed by flow cytometry.

### Cell viability assay

Mammospheres were dissociated to single cells by incubation for 3 minutes with trypsin-EDTA solution (Beit HaEmek Biological Industries, Israel), at 37°C, and then suspension with complete medium by repeated pipetting. Cells were centrifuged at 1200 rpm for 5 min, and trypsin-containing medium was discarded. Cells were resuspended with the appropriate medium, plated as triplicates in 96-well plates, at a density of 5,000 cells/well, and allowed to adhere overnight. Various concentrations of drugs were added. Control cultures received medium containing the highest concentration of the vehicle (DMSO) present in any treatment group. Plates were incubated at 37°C. Cell cytotoxicity was measured by the Neutral Red viability assay [18] or by the MTT viability assay (Alfa Aesar, USA). The cytotoxicity values of each treatment were calculated.

### Nuclear protein extracts preparation

Nuclear extracts for topoisomerase assays and Western blot analysis were prepared as described before [19, 20], except that a mixture of protease inhibitors (final concentrations: 2 μg/ml aprotinin, 2 μg/ml leupeptin, 1 μg/ml pepstatin A, 2 μg/ml antipain, 100 μg/ml PMSF) was added to the extraction buffers. Total protein concentration was determined by the BIO-Rad protein assay kit.

### Topoisomerase I activity assay

Equal quantities of purified calf thymus topo I or nuclear proteins from drug-treated and untreated cell lines were added to a topo I specific reaction mixture containing, at a final volume of 25 μl: 20 mM Tris–HCl (pH 8.0), 1 mM dithiothreitol, 20 mM KCl, 10 mM MgCl_2_, 1 mM EDTA, 30 μg/ml bovine serum albumin and 300 ng pUC19 supercoiled DNA plasmid. The indicated drugs, at various concentrations, were added to the reaction mixture prior to addition of the enzyme. Following incubation at 37°C for 30 min, the reaction was terminated by adding 5 μl of stopping buffer (final concentration: 1% sodium dodecyl sulfate (SDS), 15% glycerol, 0.5% Bromophenol blue, and 50 mM EDTA pH 8). The reaction products were analyzed by electrophoresis on 1% agarose gel using a TBE buffer (89 mM Tris–HCl, 89 mM boric acid, and 62 mM EDTA) at 1 V/cm, stained by ethidium bromide (0.5 μg/ml), and photographed using a short wavelength UV lamp (ChemiImager™ 5500 equipment, Alpha Inotech Corporation, CA, USA). Densitometric analysis of the results was performed using the EZQuant-Gel software and the percentage of topo I activity was calculated.

### Topoisomerase II activity assay

Equal quantities of purified topo II or nuclear proteins from drug-treated and untreated cell lines were added to a topo II specific reaction mixture containing, at a final volume of 25 μl: 50 mM Tris–HCl (pH 8), 0.5 mM dithiothreitol, 85 mM KCl, 10 mM MgCl_2_, 0.5 mM EDTA, 30 μg/ml BSA, and 300 ng pUC19 supercoiled DNA plasmid (for purified topo II enzyme) or 80 ng kinetoplast DNA (for nuclear-derived topo II). The indicated drugs, at various concentrations, were added to the reaction mixture prior to addition of the enzyme. Following incubation at 37°C for 30 min, the reaction was terminated by adding 5 μl of stopping buffer (final concentration; 1% sodium dodecyl sulfate (SDS), 15% glycerol, 0.5% Bromophenol blue, and 50 mM EDTA pH 8). The reaction products were analyzed by electrophoresis on 1% agarose gel using a TBE buffer (89 mM Tris–HCl, 89 mM boric acid, and 62 mM EDTA) at 1 V/cm, stained by ethidium bromide (0.5 μg/ml), and photographed using a short wavelength UV lamp (Chemilmager™ 5500 equipment, Alpha Inotech Corporation, CA, USA). Densitometric analysis of the results was performed using the EZQuant-Gel software and the percentage of topo II activity was calculated.

### Determination of the level of cellular proteins by western blot analysis

Equal quantities of nuclear proteins derived from adherent MCF7 cells or mammospheres were analyzed by Western blot analysis, as previously described [19, 21], using protein-specific antibodies. The immunocomplexes were detected by enhanced chemiluminescence (ECL).

## Results

### Isolation and characterization of MCF7-derived cancer stem-like cells

**Figure 1:**
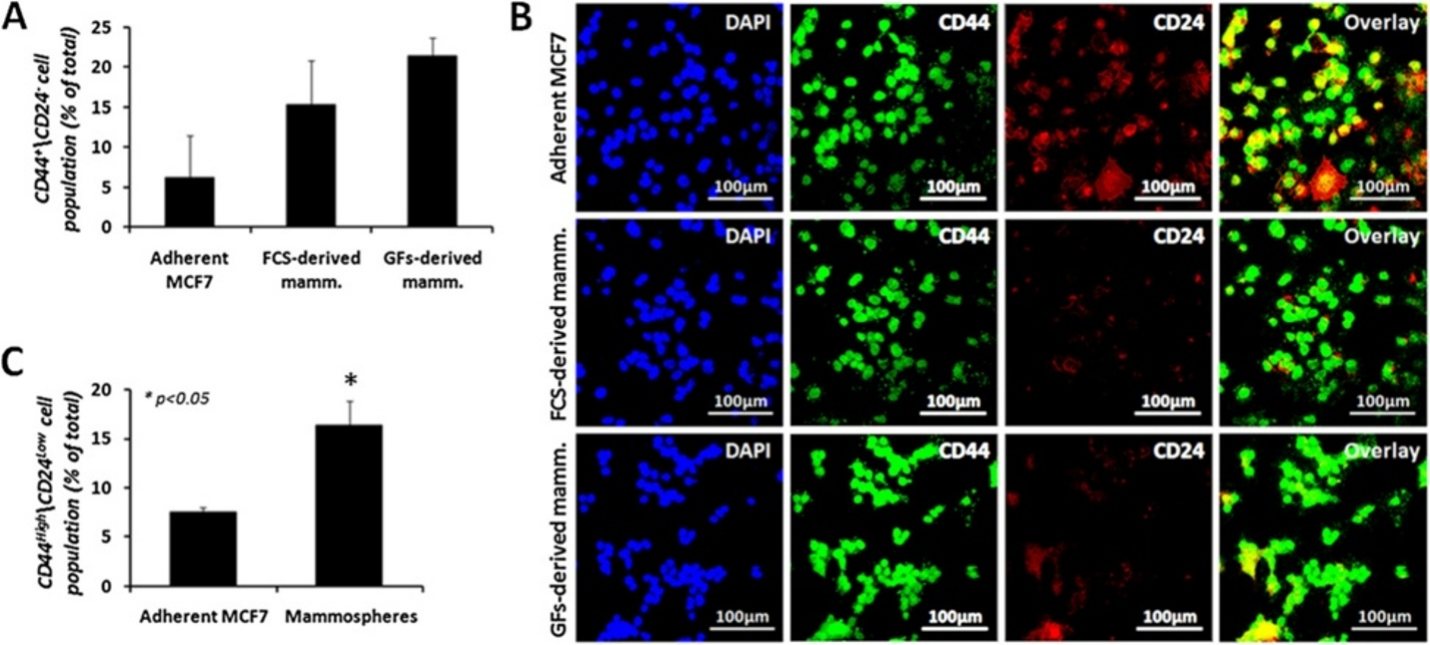
**CD44** ^**High**^/**CD24** ^**Low**^ **breast stem cell marker**-**expressing cell population is enriched in mammospheres.** MCF7-derived mammospheres were collected after 7 days in culture and dissociated. Adherent MCF7 and mammosphere-derived cells were stained for CD44 and CD24 breast stem cell markers and analyzed by flow cytometry **(A)** and immunostaining **(B)** for the CD44^high^/CD24^low^ cell population. **(C)** Immunofluorescence depiction of CD44^high^/CD24^low^ cell population was calculated manually, after acquiring several images from each cell type and counting the number of cells expressing the appropriate markers, compared to the total cell number. Results for flow cytometry represent means ± SE of 2 different mammosphere cultures. Results for immunostaining represent means ± SE of 3 different mammosphere cultures. Statistical significance was determined by the student’s *t*-test analysis: *p < 0.05.

The isolation of cancer stem cells from tumor cells population is based on their ability to grow as spheres in non-adherent, serum-free conditions. To isolate CSC from the MCF7 cell line, adherent cells were collected and grown on low-attachment plates, in a serum-free medium containing EGF, bFGF, and insulin (designated “GFs-derived mammospheres”). To verify the enrichment of cancer stem-like population within the spheres, mammospheres were grown for 7 days, collected and dissociated to single cells using trypsin-EDTA. Cells were examined for the CD44^high^/CD24^low^ breast stem cell phenotype by immunostaining or flow cytometry. The results depicted in Figure 1A show that indeed, compared to the adherent parental MCF7 cells, the CSC population is increased in mammospheres. Flow cytometry measurement revealed that the adherent MCF7 cells contain 6.2 ± 5.3% of the CSC population, and the mammospheres display a three-fold enrichment to a level of 21.5 ± 2.2%.

Although CSCs have been shown to differentiate in the presence of serum [22, 23], we determined whether MCF7-derived CSCs might also grow as spheres in the presence of serum (designated “FCS-derived mammospheres”). Indeed, mammospheres were formed in the presence of 10% fetal calf serum (FCS), and retained this ability for several passages (Additional file 1). The enrichment in the aforementioned stem cells markers was determined by immunostaining and flow cytometry. The immunostaining results (Figure 1B, C) demonstrate that the adherent MCF7 cells contain 7.6 ± 0.4% of the CSC population, while mammospheres display a two-fold enrichment of CSC markers (*p* < 0.05), to a level of 16.4 ± 2.4%. Similarly, flow cytometry revealed that while adherent MCF7 cells contain 6.2 ± 5.3% of the CSC population, FCS-derived mammospheres display more than two-fold enrichment of CSCs, to a level of 15.4 ± 5.4% (Figure 1A).

### Topo I activity is decreased and Topoisomerase II activity is increased in MCF7-derived mammospheres

**Figure 2:**
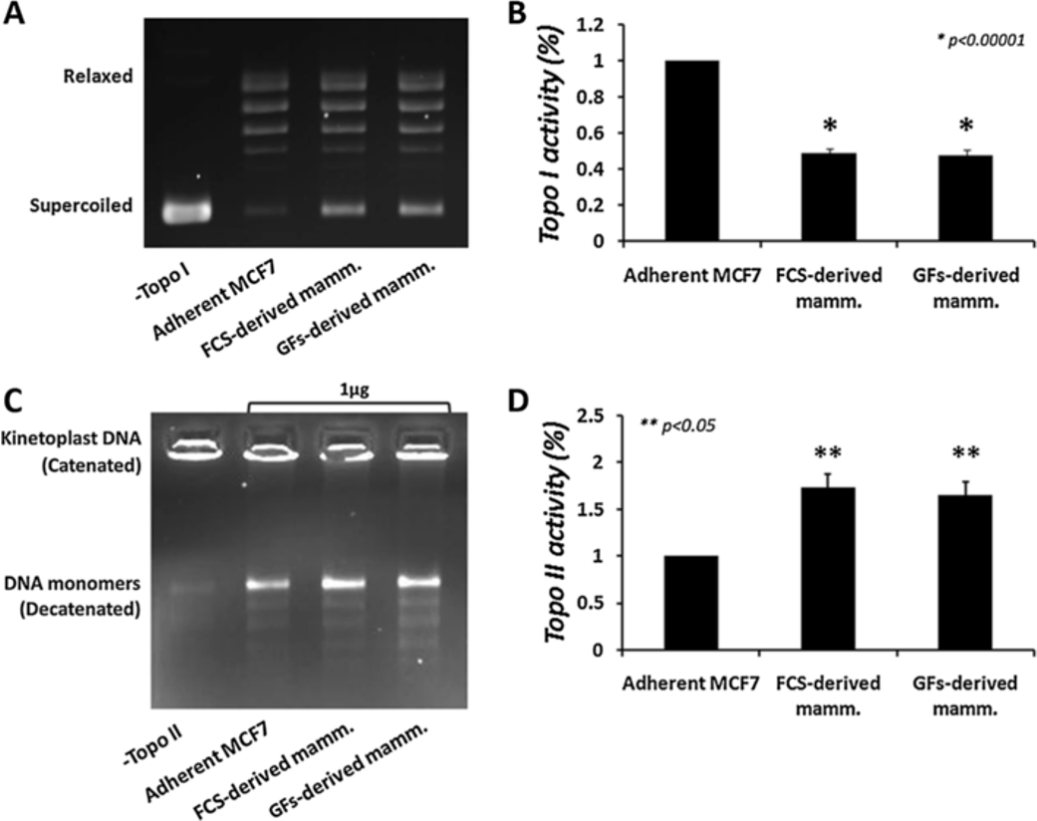
**Topoisomerases activity in mammospheres.** MCF7-derived mammospheres were isolated and nuclear extracts were made. Equal quantities of total nuclear proteins were added to a topo I- or topo II-specific reaction mixture and reaction products were analyzed by agarose gel electrophoresis. **(A)** Representative result of topo I DNA-relaxation activity. **(B)** Quantification analysis of topo I activity. **(C)** Representative result of topo II decatenation activity. **(D)** Quantification analysis of topo II activity. Results represent means ± SE of 3 experiments. Statistical significance was determined by the student’s *t*-test analysis: *p < 0.00001; **p < 0.05, compared to the adherent control.

Topoisomerases activity and expression were investigated in the CSCs-enriched mammospheres and compared to the parental adherent MCF7 cells. Mammospheres were collected and nuclear extracts were prepared. Equal protein quantities were added into a specific reaction mixture and topo I DNA relaxation activity was examined. The results depicted in Figure 2A, B show a significant decrease (*p* < 0.00001) in mammosphere-topo I activity, compared to the adherent control. Both FCS- and GFs-derived mammospheres exhibit a similar reduction, as topo I activity is reduced by 49 ± 2.4% in FCS-derived mammospheres and by 47.8 ± 2.8% in GFs-derived mammospheres, compared to the adherent cells.

It has been previously shown that topo II can compensate for topo I deficiency [24]. The decrease in topo I activity in MCF7-derived mammospheres, in comparison with the adherent cell line, led us to examine the activity of topo II in these cells. Mammospheres were collected and nuclear protein extracts were made. The parental adherent MCF7 cells served as controls. Equal proteins quantities were added to specific reaction mixtures and topo II decatenation activity was examined. The results depicted in Figure 2C, D show that topo II activity is significantly increased (*p* < 0.01) in both FCS- and GFs-derived mammospheres. Topo II activity is elevated by additional 73.7 ± 14.2% in FCS-derived mammospheres and by 65.2 ± 13.6% in GFs-derived mammospheres, compared to the adherent MCF7 cells.

### The relative levels of topoisomerase proteins are not altered in mammospheres

The changes in the activity of topoisomerases in mammospheres could be the result of (1) a change in the level of the enzyme protein or (2) posttranslational modifications that lead to modulation in activity.

**Figure 3:**
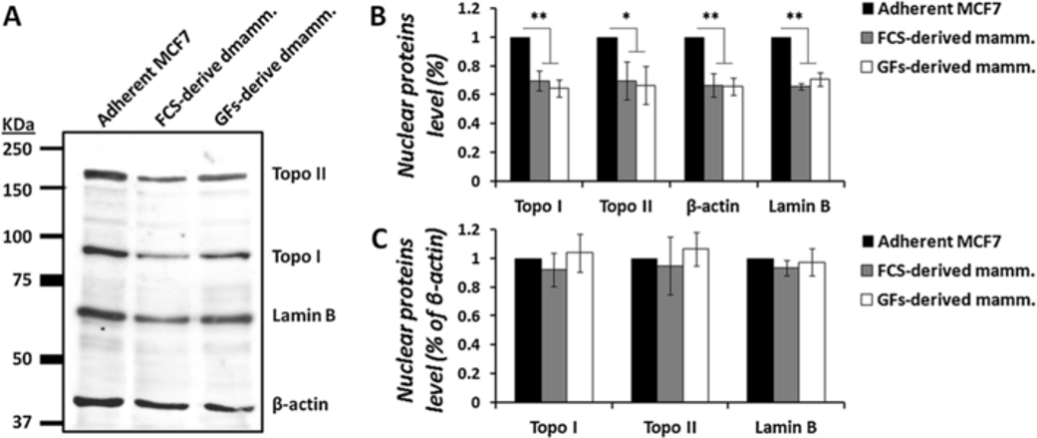
**Level of nuclear proteins in mammospheres.** MCF7-derived mammospheres were isolated and nuclear protein extracts were prepared. Equal protein quantities were examined by Western blot analysis for topo I, topo II, β-actin, and lamin B **(A)**. Densitometric analysis of the results was performed using the EZQuant-Gel software and the percentage of nuclear proteins level was calculated relative to the adherent MCF7 **(B)** or to β-actin **(C)**. Results represent means ± SE values of at least 3 experiments. Statistical significance was determined by the student’s *t*-test analysis: *p < 0.05; **p < 0.01.

To address the first possibility, topo I protein level was examined in nuclear extract proteins derived from adherent MCF7 cells and mammospheres, using equal protein quantities. As observed in Figure 3, topo I protein level was significantly decreased (*p* < 0.01) in both FCS- and GFs-derived mammospheres (by 30.1 ± 6.8% and 35.2 ± 5.8%, respectively), compared to the adherent MCF7 cells. When we examined the level of β-actin, which served as a loading control, we noticed a similar reduction (*p* < 0.01) in the protein level (33.5 ± 8.2% and 34.3 ± 6%, respectively).

In order to overcome this problem, we investigated the level of lamin B, which is abundant nuclear protein that can serve as a loading control; however a similar decrease (*p* < 0.01) was observed (34.2 ± 2.2% and 29 ± 4.8% reduction in FCS- and GFs-derived mammospheres, respectively).

Examination of topo II protein level revealed, again, a similar pattern (*p* < 0.05) of protein level reduction (30 ± 17.2% and 33.4 ± 13.3% in FCS- and GFs-derived mammospheres, respectively), despite the increased activity of the enzyme observed in mammospheres. The overall reduction in the level of nuclear proteins, which were examined in this study, could explain the reduction in topo I activity in mammospheres; however, it could not account for the elevated topo II activity in these cells. Indeed, when we examined the ratio of these proteins to β-actin levels, we observed no significant effect on the levels of proteins. Since equal amount of proteins (from all the examined samples) was added to the enzyme reaction, one may suggest that the modifications in topoisomerases activity might be the consequence of posttranslational modification of the enzyme protein.

### Topo I activity in mammospheres is regulated by poly-ADP-ribosylation

**Figure 4:**
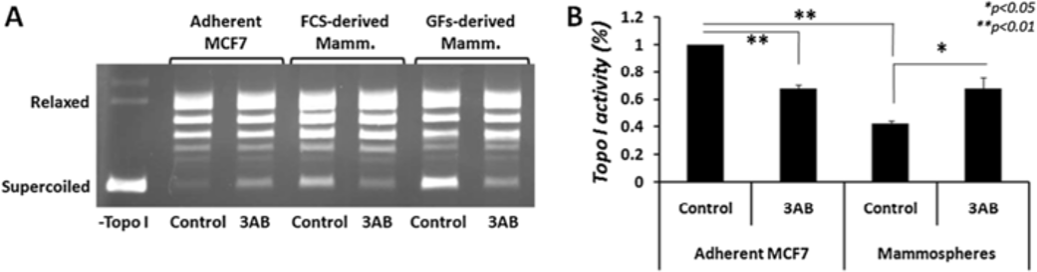
**Effect of 3**-**aminobenzamide on cellular topo I activity in mammospheres after 24 h.** Adherent MCF7 cells and mammospheres were plated at a density of 10^6^ cells\plate and treated with 3 mM 3-aminobenzamide (3AB) for 24 hours. Nuclear extracts were prepared and topo I activity was examined **(A)**. Topo I activity was quantified and plotted **(B)**. Results represent means ± SE of 3 experiments. Statistical significance was determined by the student’s *t*-test analysis: *p < 0.05; **p < 0.01.

Topo I activity is regulated by a number of posttranslational modifications [24]. One of these modifications, known to reduce topo I activity, is poly-ADP-ribosylation by the enzyme poly-ADP-ribose polymerase (PARP). To examine the possibility that PARP-1 regulates topo I activity in MCF7-derived mammospheres, we used PARP inhibitor, 3-aminobenzamide (3AB). First, the cytotoxicity of various doses (3–0.01 mM) of 3AB was examined in MCF7 cells, for up to 72 h (not shown). A slight cytotoxic effect (14.7 ± 4.8%; *p* < 0.05) was observed at 3 mM after 72 h. Since 3AB did not show marked cytotoxic effect, adherent MCF7 cells and mammospheres were treated with 3 mM of 3AB for 24 hours, and topo I activity was examined using the appropriate nuclear extracts. The results depicted in Figure 4 show that, indeed, the cellular topo I activity is increased in mammospheres after PARP inhibition (from 42 ± 2.5% to 67.4 ± 8.3%), and restored to a comparable level as observed in adherent MCF7 cells after 3AB treatment (67.6 ± 2.6%). These results indicate that topo I activity in mammospheres is regulated, at least in part, by poly-ADP-ribosylation.

### Reduction of topo I activity is a common characteristic of stem cell sphere-formation

**Figure 5:**
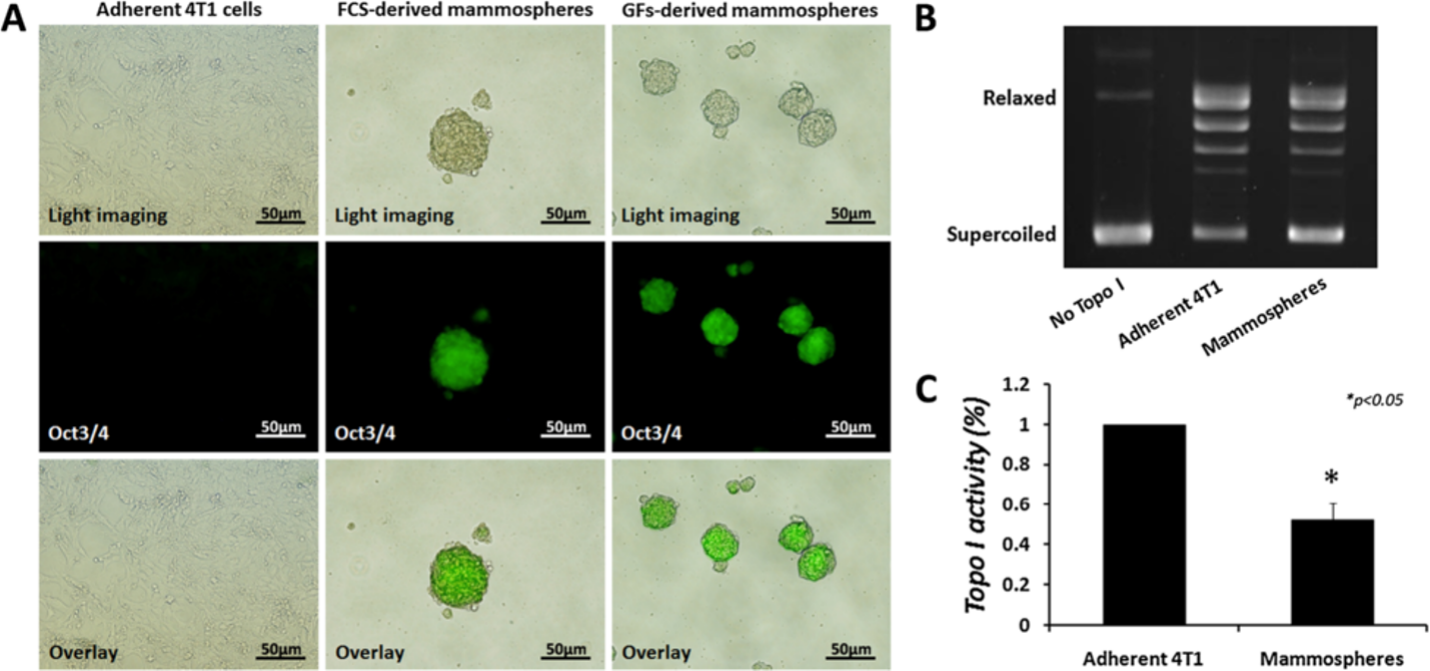
**Topo I activity in 4 T1**-**Luc**-**Oct3/** **4pG**-**derived mammospheres.** Adherent 4 T1-Luc mouse cells, expressing green fluorescent protein zsGreen under Oct-3/4 promoter, were grown as mammospheres under the conditions indicated. **(A)** Mammospheres were examined for Oct-3/4 expression by the visualization of fluorescence (green) under the same exposure setting expression. Images were obtained by fluorescent microscopy. After 6 days in culture, 4 T1-Luc-Oct3/4pG-derived mammospheres were isolated and nuclear extracts were made. Total nuclear proteins were added to a topo I-specific reaction mixture and reaction products were analyzed by agarose gel electrophoresis. **(B)** Representative result of topo I DNA-relaxation activity. **(C)** Quantification analysis of topo I activity. Results represent means ± SE of 3 experiments. Statistical significance was determined by the student’s *t*-test analysis: *p < 0.05.

To further substantiate the changes in topo I activity observed in mammospheres, we utilized other cancer stem cell systems. Adherent 4 T1-Luc mouse mammary carcinoma cells, transfected with Oct3/4 pGreenZeo promoter reporter construct (express green fluorescent protein zsGreen under the stem cell marker Oct-3/4 promoter), were grown as mammospheres, as mentioned before. Cells were examined for Oct-3/4 expression by the visualization of fluorescence, under the same exposure setting. Figure 5A demonstrates increased expression of Oct3/4 stem cell marker (green) in mammospheres, compared to the parental adherent 4 T1-Luc-Oct3/4pG cells, indicating an enrichment of cancer cells with stem-like properties.

After the establishment of mammospheres’ stem-like properties, adherent 4 T1-Luc-Oct3/4pG cells and their derived mammospheres were collected, nuclear protein extracts were prepared, and topo I activity was examined. The results depicted in Figure 5B and C show that in mouse mammary CSCs, similarly to human cells, topo I activity is reduced (*p* < 0.05) by 47.5 ± 8.5%, compared to the parental adherent cell line.

**Figure 6:**
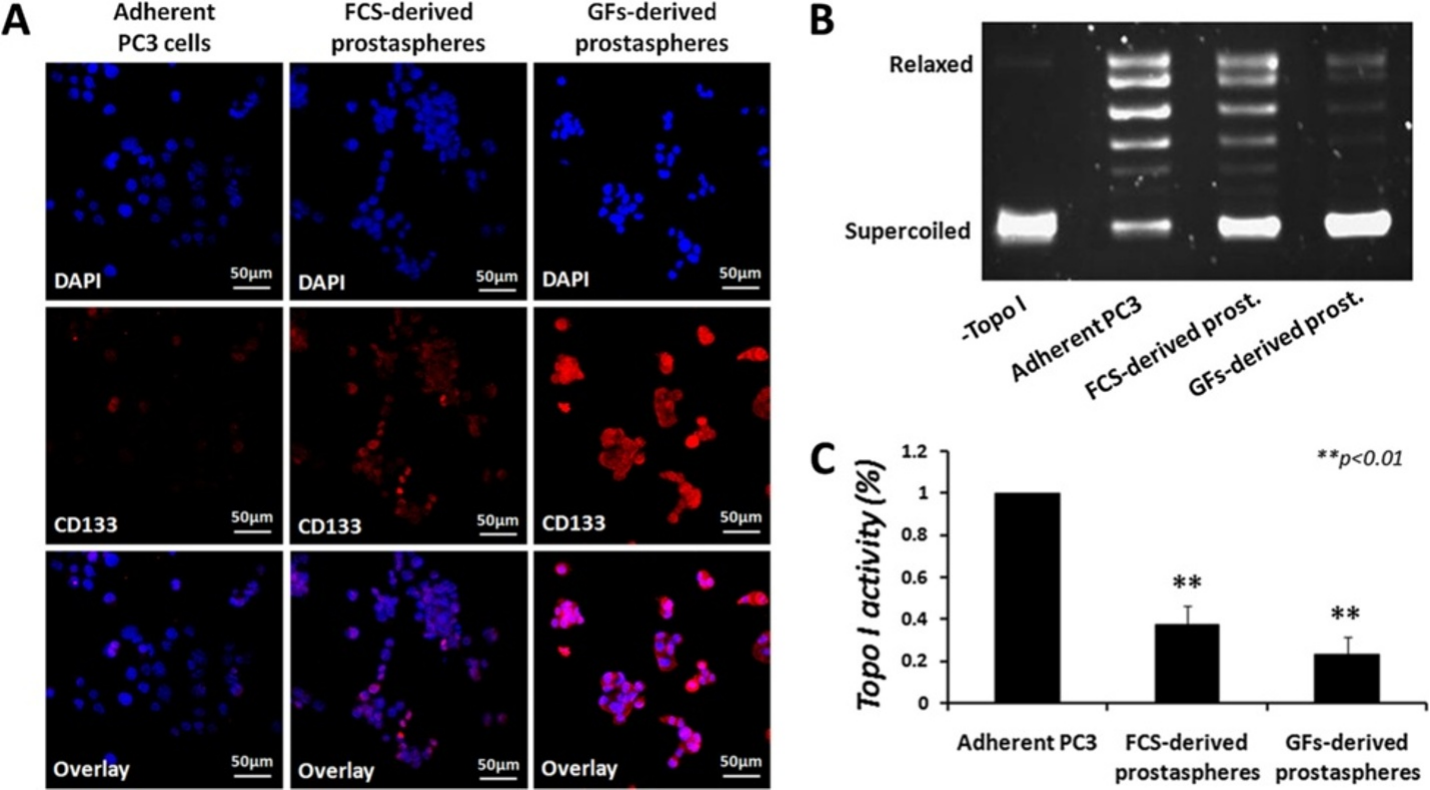
**Topo I activity in PC3**-**derived prostaspheres.** PC3-derived spheres were collected and dissociated enzymatically to single cells. **(A)** Adherent PC3 and prostasphere-derived cells were stained for CD133 stem cell marker and analyzed by immunostaining. Results represent 3 different prostasphere cultures. PC3-derived prostaspheres were isolated and nuclear extracts were made. Equal quantities of total nuclear proteins (25 ng) were added to a topo I-specific reaction mixture and reaction products were analyzed by agarose gel electrophoresis. **(B)** Representative result of topo I DNA-relaxation activity. **(C)** Quantification analysis of topo I activity. Results represent means ± SE of 3 experiments. Statistical significance was determined by the student’s *t*-test analysis: **p < 0.01, compared to the adherent control.

Furthermore, we utilized the sphere formation assay to isolate prostate CSCs from the human PC3 cell line, in both FCS- and GFs-supplemented media. PC3-derived spheres (termed “prostaspheres”) were collected and examined for expression of stem cell marker CD133 by immunostaining. The results depicted in Figure 6A show that prostaspheres express higher levels of CD133 than the parental adherent cells, indicating stem cell enrichment.

After the establishment of stem-like properties, prostaspheres were collected and nuclear protein extracts were prepared. Topo I activity was examined and compared to the parental adherent PC3 cells. The results depicted in Figure 6B and C show that in accordance with the aforementioned observations, compared to the parental adherent cells, prostaspheres exhibit decreased topo I activity (*p* < 0.01), as FCS-derived spheres show a 62 ± 8.4% decrease and GFs-derived spheres show a 76.3 ± 8.3% decrease.

**Figure 7:**
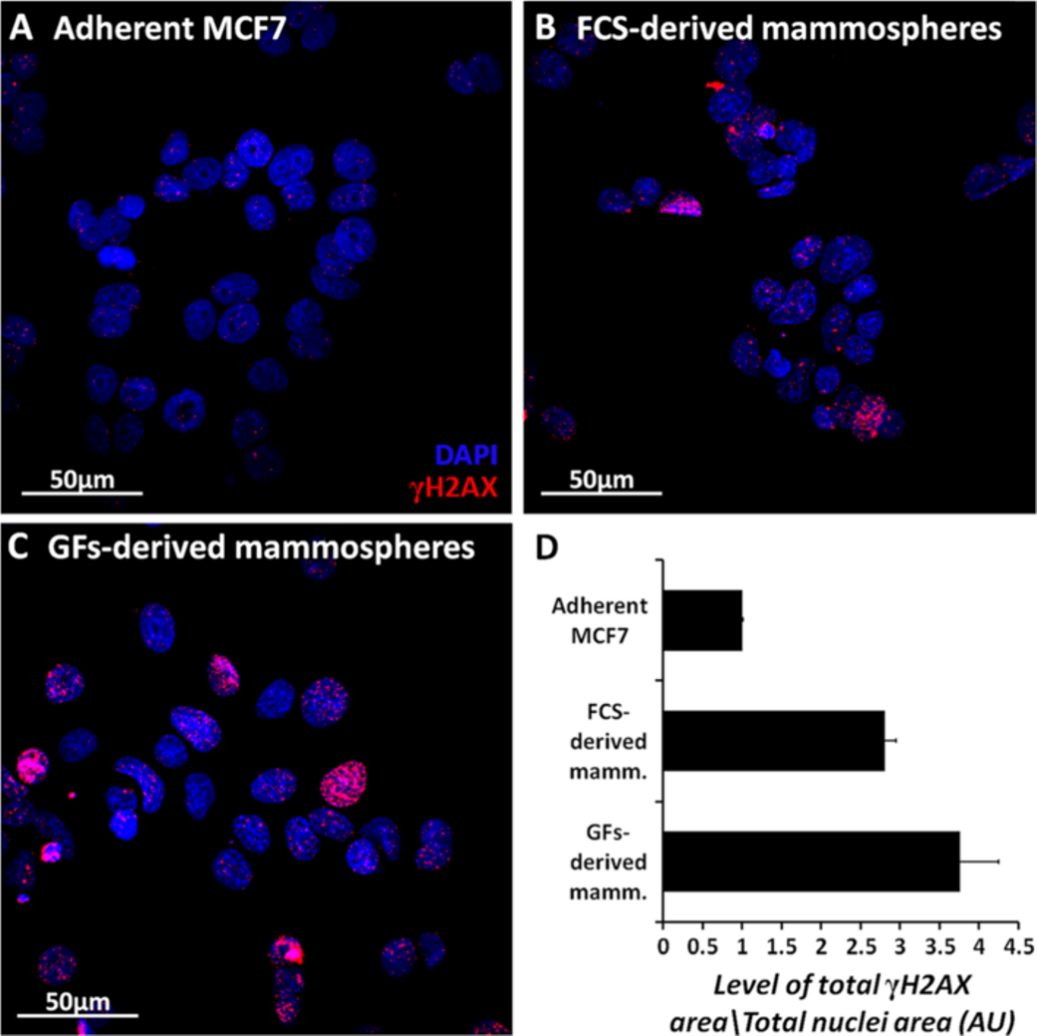
**Levels of** **γH2AX in adherent MCF7 cells and mammospheres.** Adherent MCF7 cells **(A)** and mammosphere-derived cells **(B, C)** were seeded onto Lab-Tek chamber slides and allowed to adhere overnight. Cells were fixed using methanol:acetone and stained for γH2AX (red). Nuclear staining was achieved by DAPI (blue). Images were obtained by confocal microscopy. Level of γH2AX staining area was measured compared to total nuclear area and plotted **(D)**. Images represent 3 different mammosphere cultures.

The regulation of stem cell self-renewal and differentiation has been shown to induce chromatin remodeling processes. It has been demonstrated that mouse embryonic stem cells (mESCs) and mouse iPS cells have high basal levels of γH2AX, in a mechanism that is not dependent on DNA damage response. Levels of γH2AX have been shown to decrease upon differentiation, and increase in the presence of self-renewal-enhancing molecules [25]. Here we examined γH2AX levels in MCF7-derived mammospheres by the immunostaining assay. The results depicted in Figure 7 show that γH2AX levels (red) are elevated in mammosphere-derived cells, compared to the adherent MCF7 cells, suggesting self-renewal regulation processes.

### Mammosphere-derived cells are resistant to topo I inhibition by CPT or TPT and sensitive to topo II inhibition by etoposide

Since CPT (and its derivatives) and etoposide exert their inhibitory effect on DNA-bound topoisomerases, i.e., active topoisomerases, reduced topo I activity, as observed for mammospheres, is expected to result in the reduction of total DNA bound-topo I, rendering reduced sensitivity to CPT and its derivatives. Similarly, elevated topoII activity, as observed for mammospheres, is expected to result in the increase of total DNA bound-topo II, rendering increased sensitivity to etoposide. Therefore, MCF7 cells were grown as mammospheres for 7 days and then dissociated enzymatically, using trypsin-EDTA. Cells were seeded as triplicates in a 96-well plate at a density of 5,000 cells/well, in the presence of 1% serum, to allow proper attachment. Cells were treated with CPT, topotecan (also termed TPT – a CPT derivative currently in use as an anti-cancer drug [9]), and etoposide for 24 hours; cell viability was measured by the Neutral red cytotoxicity assay.

**Figure 8:**
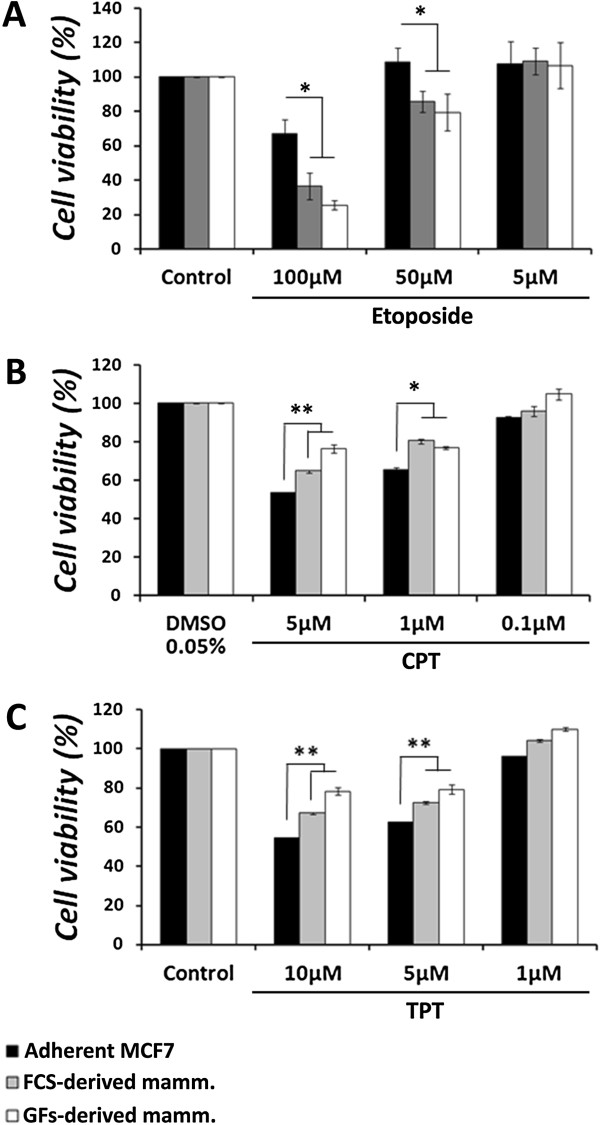
**Effect of topoisomerase inhibitors on mammosphere**-**derived cells for 24 h.** MCF7 cells were grown in non-adherent conditions. After 7 days mammospheres were dissociated for single cells and plated in 96-well plates at a density of 5,000 cells/well, in the presence of 1% serum. Cells were treated with various concentrations of etoposide **(A)**, CPT **(B)** or TPT **(C)** for 24 h. Cell viability was examined by the neutral red cytotoxicity assay and growth inhibition was calculated, compared to the control of each cell type. Results represent means ± SE of 3 experiments. Statistical significance was determined by the student’s *t*-test analysis: *p < 0.05; **p < 0.01.

The results depicted in Figure 8A show that the adherent MCF7 cells are significantly more resistant to etoposide treatment, compared to cells derived from mammospheres. Etoposide, at 100 μM, reduced the viability of adherent cells by 33.1 ± 7.7%, while the reduction of cell viability in mammosphere-derived cells was 63.5 ± 9.3% in FCS-derived mammospheres and 74.7 ± 1.7% in GFs-derived mammospheres (*p* < 0.05). It is notable that the adherent cells showed remarkable recovery at doses lower than 100 μM, whereas the mammosphere-derived cells did not. While MCF7 cells showed complete resistance to 50 μM of etoposide, significant decrease in cell viability (*p* < 0.05) of 14.2 ± 8.2% was observed for FCS-derived mammosphere cells and 20.5 ± 10.7% for GFs-derived mammosphere cells.

In contrast, the adherent MCF7 cells showed significantly higher sensitivity to CPT treatment, compared to cells derived from mammospheres (*p* < 0.05). The results depicted in Figure 8B show that CPT, at 5 μM to 1 μM, caused 46.9% to 34.8 ± 1.5% reduction (respectively) in the viability of the adherent cells, while in the FCS-derived mammosphere cells CPT treatment showed 35.4 ± 0.7% to 19.8 ± 1.2% decrease in cell viability (a 23.3–11.6% difference), and in the GFs-derived mammospheres it exhibited a 23.6 ± 2.4% to 23.2 ± 0.8% decrease in cell viability (22–8% difference).

In addition to CPT, we examined the sensitivity of the cells to another topo I inhibitor – topotecan (TPT), a water soluble derivative of CPT. As depicted in Figure 8C, significant resistance was observed with TPT treatment in mammosphere-derived cells, compared to the adherent cells (*p* < 0.05). TPT, at 10 μM to 5 μM, respectively, caused 45.8 ± 0.32% to 37.7 ± 0.56% reduction in the viability of adherent cells, while FCS-derived mammosphere cells showed32.9 ± 0.4% to 27.5 ± 0.7% reduction in cell viability (12.9–10.2% difference), and GFs-derived mammospheres showed 21.7 ± 2.1% to 20.8 ± 2.2% (24.1–16.9% difference) reduction in cell viability.

### Intact mammospheres exhibit increased chemoresistance to topoisomerases inhibition, by both CPT and etoposide, compared to the adherent MCF7 cells

**Figure 9:**
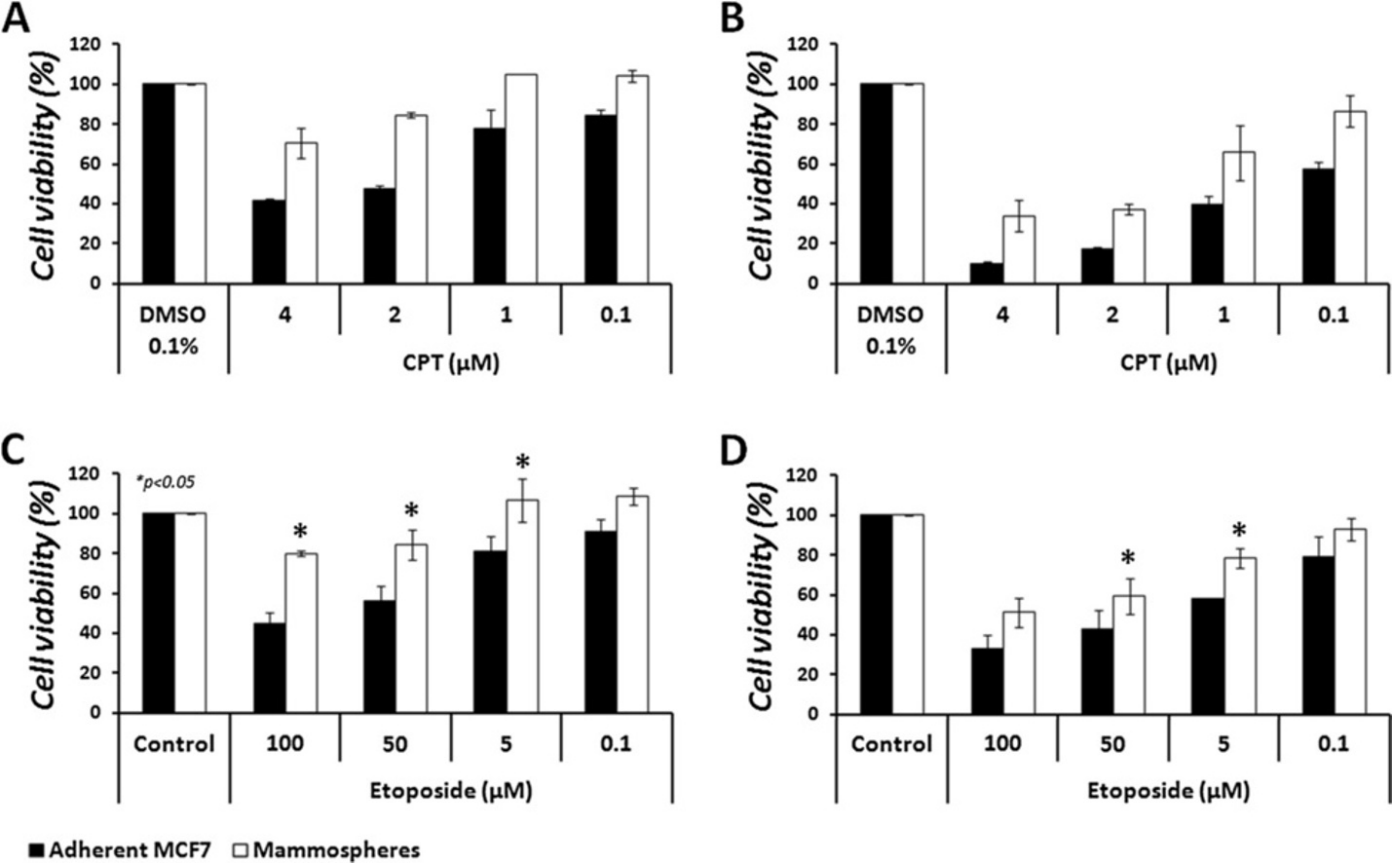
**Cytotoxic effect of CPT and etoposide on intact mammospheres.** Mammospheres were collected after 5–7 days in culture and total number of cells was evaluated. Adherent MCF7 cells and intact mammospheres were seeded in 96-well plates at a density of 5,000 cells/well. The parental MCF7 cells were allowed to adhere over night, after which cells were treated with various doses of CPT **(A, B)** or etoposide **(C, D)** for 24 **(A, C)** or 48 hours **(B, D)**. Cell viability was examined by the MTT viability assay. Results for CPT represent means ± SE of 2 experiments. Results for etoposide represent means ± SE of 3 experiments. Statistical significance was determined by the student’s *t*-test analysis: *p < 0.05.

In the above described experiments, the sensitivity of cell suspensions derived from mammospheres to topoisomerase inhibitors was determined. However, since the structure of a mammosphere may interfere with the entrance of the drugs, we investigated the sensitivity of the intact mammospheres to topoisomerase antagonists alone, as a single drug, or in combination with other anti-cancer drugs. Mammospheres were grown for 5–7 days, collected, and total number of cells was evaluated. Mammospheres were seeded in low-attachment 96-well plates and treated with various combinations of the indicated drugs for up to 48 hours. Cell viability was examined every 24 hours by the MTT viability assay. The results depicted in Figure 9 show that, as expected, CPT exhibits reduced cytotoxicity in mammospheres compared to the adherent cells (A, B). However, unexpectedly, etoposide also showed significant (*p* < 0.05) reduced cytotoxicity in mammospheres (C, D). This was not compatible with the elevated topo II activity (see Figure 2), and the sensitivity of mammosphere-derived cells (see Figure 8).

**Figure 10:**
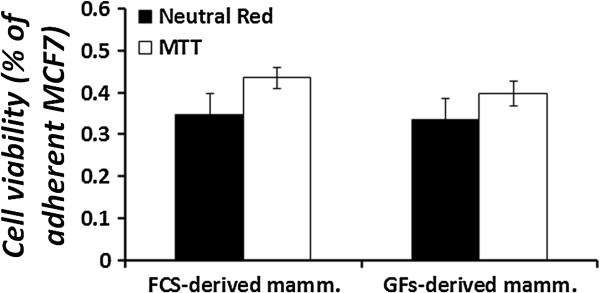
**Mammosphere cell viability.** Adherent MCF7 cells and mammospheres were plated as triplicates in 96-well plates, at a density of 5,000 cells/well and allowed to grow. Cell viability was determined every 24 hours by the MTT viability assay (black bars). Alternatively, adherent MCF7 cells and mammospheres were cultured at a density of 10^6^ cells\plate and allowed to grow. Cells were collected every 24 hours and suspended with 1 ml of complete medium, after which 100 μl were transferred to 96-well plates, in triplicates. Cells were allowed to adhere overnight and cell viability was evaluated by the Neutral Red viability assay (white bars). Results represent means ± SE of at least 3 experiments. Statistical significance was determined by the student’s *t*-test analysis, compared to the adherent control.

To examine the possibility that this reduced sensitivity is affected by the inability of MTT to enter the bulk of cells in mammospheres, rather than the cytotoxic effects of the indicated topoisomerase inhibitors, intact and dissociated mammospheres were grown on 96-well plates, without treatment. After 24 hours in culture, cell viability was examined by either MTT (for intact spheres) or Neutral red (for dissociated single cells, grown as monolayer) assays. As depicted in Figure 10, cell viability was not significantly different for both dissociated and intact mammospheres, suggesting that the cellular structure does not influence the indicated viability assay, and that these assessments are appropriate. Thus, the differed sensitivities for CPT and etoposide shown in Figures 8 and 9, probably stem from active cellular mechanisms, obtained by the sphere formation process.

### A combined treatment of CPT with Gefitinib or etoposide with Erlotinib shows increased cytotoxic effect in mammospheres

**Figure 11:**
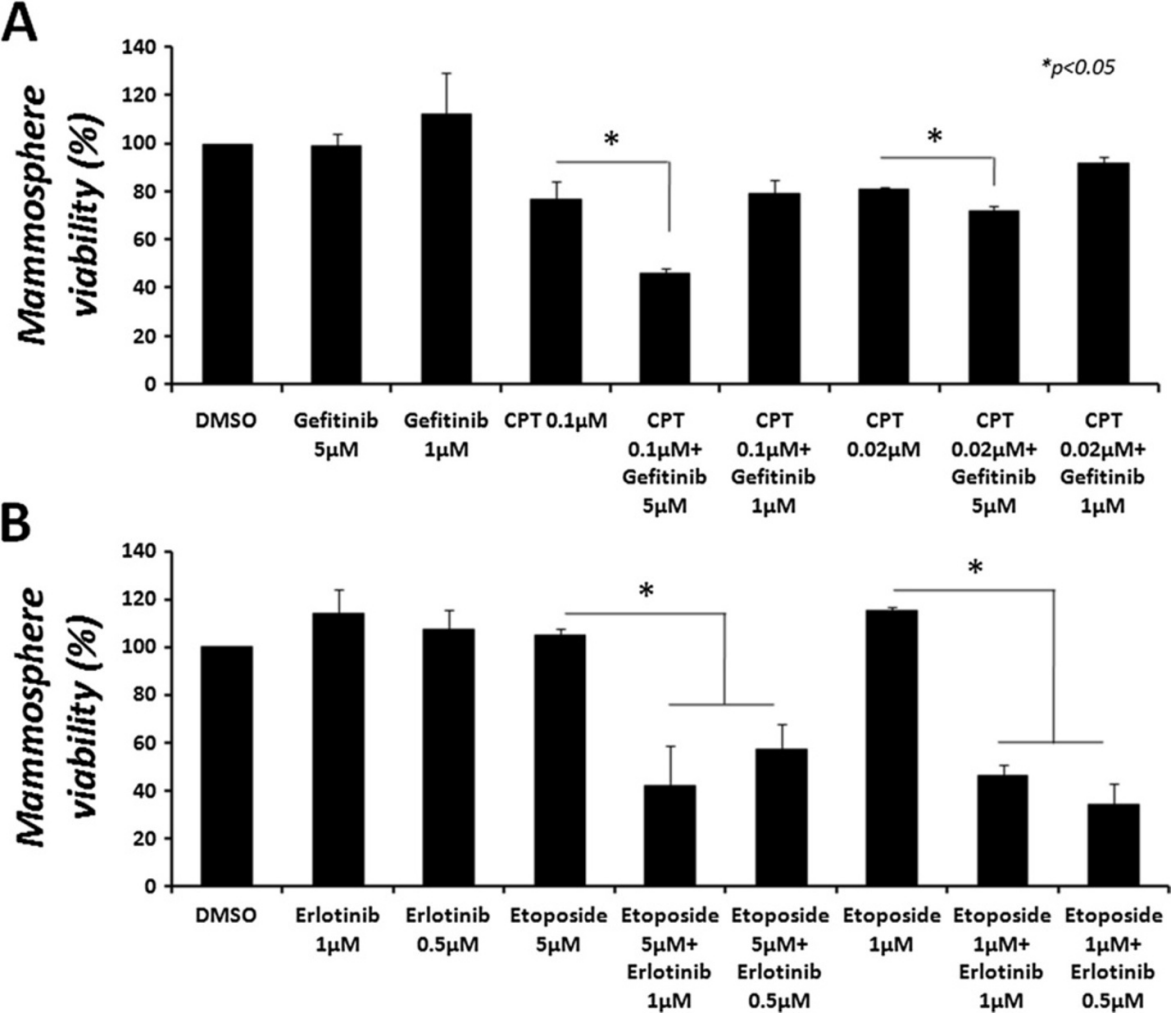
**Cytotoxic effect of a combined treatment with CPT and Gefitinib or etoposide and Erlotinib on intact mammospheres.** Mammospheres were collected after 5–7 days in culture and total number of cells was evaluated. Intact mammospheres were seeded in low-attachment 96-well plates at a density of 5,000 cells/well, after which cells were treated with **(A)** the combination of CPT (0.1 μM or 0.02 μM) and Gefitinib (5 μM or 1 μM), or **(B)** the combination of etoposide (5 μM or 1 μM) and Erlotinib (1 μM or 0.5 μM) for 72 hours. Cell viability was examined by the MTT viability assay. Results represent means ± SE of 2 experiments. Statistical significance was determined by the student’s *t*-test analysis: *p < 0.05.

Previous studies have demonstrated an increased anti-cancer effect of the combination of topoisomerase inhibitors and tyrosine kinase antagonists [14–17]. The chemoresistance of mammospheres to topoisomerases inhibitors led us to investigate the efficacy of their combination with TKIs – Erlotinib and Gefitinib – at low drug concentrations. Mammospheres were collected after 5–7 days in culture and total number of cells was evaluated. Mammospheres were seeded in low-attachment plates and treated with various combinations of the indicated drugs, for up to 72 h. Mammosphere viability was examined by the MTT viability assay. No significant effects were observed for up to 48 h in the combined treatments, compared to the monotherapeutic effects of CPT or etoposide (not shown). Note that neither Erlotinib nor Gefitinib showed significant cytotoxicity as single agents at the doses examined, for up to 72 h (not shown). However, both Erlotinib and Gefitinib were able to potentiate the cytotoxic effect of topoisomerase inhibitors 72 hours post-treatment, as depicted in Figure 11. Figure 11A demonstrates that Gefitinib, at 5 μM, significantly potentiated the cytotoxic effect of CPT (*p* < 0.05). While CPT, at 0.1 μM, showed a cytotoxic effect of 22.9 ± 7.4%, the combined treatment increased this effect to 53.9 ± 2.1%. At 0.02 μM CPT, Gefitinib was only able to increase the cytotoxic effect by 9.3%, from 18.8 ± 0.8% for CPT alone to 28.1 ± 2% in the combined treatment.

Similarly, Figure 11B demonstrates that Erlotinib, at 1 μM and 0.5 μM, significantly potentiated the cytotoxic effect of etoposide (*p* < 0.05). While both Erlotinib and etoposide, at either 5 μM or 1 μM, showed no cytotoxic effect, the combined treatment revealed a striking increase in cytotoxicity. At a concentration of 1 μM, Erlotinib increased the cytotoxicity of etoposide to a level of 42.1 ± 16.5% and 46.4 ± 4.9% viability for 5 μM and 1 μM of etoposide, respectively. At a concentration of 0.5 μM, Erlotinib increased the cytotoxicity of etoposide to a level of 57.2 ± 10.8% and 34.1 ± 9.3% viability for 5 μM and 1 μM of etoposide, respectively.

## Discussion

In the past few decades there has been accumulating evidence suggesting that tumors are generated by a subset of rare cells, expressing stem/progenitor cell characteristics. These cells, termed “cancer stem cells” (CSCs), have been suggested to account for tumor growth, progression, metastasis, and cancer relapse, as they display high tumorigenicity in animal models and show increased resistance to chemo- and radio-therapy [1, 4, 5, 8, 26].

Since topoisomerases are essential nuclear enzymes for DNA transactions, we characterized their activity in mammospheres derived from the MCF7 breast cancer cell line. This model has been widely studied for evaluation of CSC biology, and here we utilized it as a model for the evaluation of breast CSC response to topoisomerases inhibitors, CPT and etoposide, and the combined treatments based on these drugs with tyrosine kinase inhibitors (TKIs). For this purpose, mammospheres were examined for topoisomerases activity in differentiating (10% FCS) and non-differentiating (Growth Factors – GFs) conditions and CD44^+^/CD24^−^ breast stem cell markers were verified. Indeed, mammospheres were enriched for the CD44^+^/CD24^−^ stem cell population, as established by immunostaining and FACS analysis.

Since both FCS- and GFs-derived mammospheres exhibited similar behavior throughout this study, they will not be distinguished in this section.

Examination of topoisomerases activity reveals that, in comparison with the parental adherent MCF7 cell line, mammospheres exhibit reduced topo I activity. Conversely, topo II activity is increased in mammospheres. When we examined topoisomerases protein levels we did not observe changes relative to the loading controls, β-actin and lamin B; however, an overall reduction was observed in all four proteins. It has been previously shown that changes in cell morphology and culture adherence status lead to altered expression of β-actin [27]. Embryonic chicken fibroblasts, treated with Phorbol-12-myristate-13-acetate (PMA), were found to create foci of densely packed cells that stopped adhering to the plates and had reduced levels of β-actin mRNA and protein. In contrast, floating chondrocytes treated with PMA adhered to the culture dish and displayed increased β-actin mRNA and protein levels [27]. Since the process of CSC isolation involves cell growth in floating conditions and results in the formation of seemingly packed spheres, it is possible that this morphology leads to the observed reduction in β-actin protein levels.

In addition to decreased β-actin, lamin B is also reduced in mammospheres. It has been reported that while lamin B is not needed for proper self-renewal and pluripotency of embryonic stem cells, lamin B interactions with chromatin are increased after differentiation, thereby suppressing gene transcription, showing their importance in lineage determination [28]. Lamin B has also been shown to be involved in the formation of nuclear skeletal support [29], which might also be modified due to structural changes during mammosphere formation, similarly to β-actin. Lamin B has been shown to be involved in replication, as it localizes with the replication factor proliferating cell nuclear antigen (PCNA) in the replication machinery during late S phase [29]. Since mammospheres exhibit a low cycling phenotype, the reduced level of lamin B might also be a mechanism of the mammospheres, as shown by the cell viability assays.

Although an overall reduction was observed in the level of nuclear proteins, at least for the indicated peptides, the relative level of topo I enzyme protein was not changed in mammospheres, compared with the adherent MCF7 cells. When we examined the sensitivity of the enzyme to topo I inhibitor CPT, an expected resistance was observed. This prompted us to investigate a possibility that topo I protein might be altered in mammospheres, translationally (i.e., alternative splicing or folding) or post-translationally. To examine this possibility, topo I activity levels of the adherent-derived and mammosphere-derived enzymes were normalized *in vitro*. CPT sensitivity was examined and revealed a similar inhibition level in both cell types (data not shown), suggesting that it is not a translational, but a posttranslational modification.

Topo I has been shown to be regulated by poly-ADP-ribose polymerase 1 (PARP-1). While poly-ADP-ribosylation by PARP-1 reduces the activity of topo I, a direct interaction between the two enzyme proteins leads to topo I activation [24, 30–32]. Since topo I activity is reduced in mammospheres, PARP-1 poly-ADP-ribosylation activity was inhibited in mammospheres by 3-aminobenzamide (3AB). Indeed, topo I activity was restored to a similar level as that of adherent MCF7 cells treated with 3AB, indicating that topo I is regulated, at least in part, by PARP-1.

PARP-1 is a ubiquitous nuclear enzyme that is activated in response to DNA damage [33]. The observation that topo I is regulated by PARP-1 in mammospheres, lead us to investigate the possibility that the reduction in topo I activity might result from DNA damage-induced PARP-1 activation. For this purpose, we examined the formation of phosphorylated histone 2AX (γH2AX) foci in the nucleus, which are a hallmark of DNA breaks [25]. Indeed, mammospheres exhibit increased levels of γH2AX, compared to the adherent MCF7 cells.

Although increased γH2AX can be regarded as DNA damage, it has been shown that γH2AX induction can be a result of chromatin remodeling, a process that has been characterized during stem cell renewal and differentiation, without activation of DNA damage response [25]. Indeed, PARP-1 is also involved in the process of chromatin remodeling [34]. Furthermore, topo I has been shown to be reduced in differentiated cells; however, it has also been shown to be lower in non-proliferating cells, compared to log-phase proliferating cells [35], which might point to several processes that regulate topo I activity within the sphere.

These questions led us to investigate whether the reduction in topo I activity is a global characteristic of cancer stem cells. CSCs were isolated as spheres from various cell types, including mouse mammary 4 T1-Luc tumor cells that express zsGreen fluorescent protein under the Oct3/4 promoter, and the PC3 human prostate cancer cells. Mammospheres derived from 4 T1-Luc-Oct3/4pG cells were examined for Oct3/4 expression, as an indicator of stem properties, since Oct 3/4 transcription factor is a known stem cell marker, which was shown to be critical for maintaining stem cell characteristics [36]. PC3-derived prostaspheres were examined for the expression of stem cell marker CD133.

After establishment of stem-like properties, 4 T1-Luc-Oct3/4pG- and PC3-derived spheres were examined for topo I activity. Similar results were obtained for both cell types, as topo I activity was reduced in comparison to the parental adherent cells, indicating a global tumorsphere property. It has been shown that Oct3/4-expressing cancer stem-like cells (express GFP under Oct3/4 promoter), isolated from breast cancer cell lines, and CD49f^+^ primary cancer cells exhibit higher topo I activity and protein level, and display increased sensitivity to topo I inhibitors [37]. In contrast CD44^+^ stem-like cells, isolated from colorectal cancer cell line, displayed reduced topo I activity and insensitivity of the enzyme to camptothecin, compared to the CD44^−^ subpopulation [38]. In both studies tumor spheres were not isolated as described in this manuscript. Mammospheres can be generated by single cells, as we also observed in both FCS and GFs-supplemented media (not shown), and yet display a heterogenic progenitor composition [22]. Although sphere assays are being extensively used to evaluate stem cell activity in normal tissue and CSCs, in the majority of these cultures there is no definitive information as to which cells are being propagated. The characteristics of spheres and their relationship with their stem cells have been unclear, causing over-interpretation of results in many cases. This is largely because extensive self-renewal has been difficult to define in the context of a sphere assay [8]. It is possible then, that along with self-renewal of CSCs, differentiation processes can take place. Indeed, mammospheres only exhibit approximately 15–20% of the CD44^+^/CD24^−^ stem cell population (as observed by FACS analysis); thus the specific characteristics of single CSCs are not possible under these conditions. All together these observations suggest that topo I activity and expression in CSCs might be influenced by the sphere structure and composition.

Since mammospheres exhibit a relatively low cycling rate and no relative changes in the level of topo II protein, chromatin remodeling during self-renewal and differentiation processes within the sphere might also explain the increased topo II activity, as the enzyme is necessary for this process. An alternative explanation for topo II increased activity could be a compensation mechanism, caused by the reduced topo I activity. Although topo II has been mostly shown to compensate for topo I reduction after treatment with CPT, as observed by increased levels of the topo II protein [39], the reduction in topo I activity by a posttranslational modification, rather than protein downregulation, might similarly lead to increased topo II activity. Indeed, it has been shown that topo II compensates for topo I deficiency in a stable siRNA model. Moreover, this compensation correlated with increased γH2AX foci, chromosomal rearrangement, and chromatin remodeling activities [40].

Since topo I showed reduced activity and consequently increased resistance to CPT *in vitro*, we examined the sensitivity of mammosphere-derived topo II to etoposide. As expected, topo II showed increased sensitivity to etoposide.

The response of topoisomerases to their corresponding inhibitors, in correlation with their activity *in vitro*, prompted us to examine the sensitivity of mammospheres to CPT and etoposide. First we examined the sensitivity of mammosphere-derived cells to these drugs. Again, cells expectedly displayed an increased resistance to CPT, correlated with the reduced topo I activity. In addition, mammosphere-derived cells exhibit increased sensitivity to etoposide, which correlates with the increased topo II activity. These results are compatible with a previous observation in an epithelial-mesenchymal transition (EMT) model of breast CSC, in which anti-cancer agents were screened by their CSC-specific toxicity, revealing increased cytotoxicity for etoposide and reduced cytotoxicity for CPT [41].

Next, we examined the sensitivity of intact mammospheres to topoisomerases inhibitors. Surprisingly, while mammospheres exhibit increased resistance to CPT, as expected, this effect was also observed for etoposide. It has been demonstrated that CSCs express high levels of ABC transporters, as this characteristic enables their identification by the “dye efflux” assay. It is known that etoposide is a substrate for MDR protein [42]. The contradicting results shown for etoposide in mammosphere-derived cells and intact mammospheres could be explained by the fact that the Neutral red cytotoxicity assay performed with mammosphere-derived cells requires cells to adhere to the culture plate. The heterogeneous composition of mammosphere cells might indicate that not all cells within the sphere express MDR transporters. It has been shown that mammospheres of a tamoxifen-resistant MCF7 cell line exhibits increased breast cancer resistance protein (BCRP) levels in response to tamoxifen treatment. The expression of BCRP was mostly expressed in the outer cells of the mammosphere [43]. It is possible that the cellular interactions and compartments within the sphere might allow for proper protection of the inner mass of the sphere after exposure to anti-cancer drugs, thus enabling efficient ATP management. This characteristic could be lost after sphere dissociation and changes in growth conditions. It is notable that CPT is a poor substrate for MDR [44]; thus the reduced sensitivity in mammospheres is mostly due to the reduction of topo I activity.

When we examined the cytotoxicity of the combined treatments we observed that Gefitinib increased the anti-cancer effect of CPT in mammospheres; however Erlotinib failed to show a significant beneficial effect in the combined treatment, compared to each drug administered alone, in a preliminary examination. While mammospheres were resistant to etoposide, the addition of Erlotinib dramatically increased its cytotoxic effect. Combination with Gefitinib, however, showed no significant beneficial effect in the combined treatment with etoposide, compared to each drug administered alone in a preliminary examination.

Several studies have shown that both Gefitinib and Erlotinib can modulate the activity of ABC transporters and invert resistance of cancer cells [45–48]. While the ability of Erlotinib to increase the anti-tumor effect of etoposide in mammospheres could be attributed to its potential to inhibit MDR proteins, it could also exert its anti-EGFR and anti-topoisomerase activity [17]. However, since CPT is a poor substrate for MDR transporters, Gefitinib was able to increase its cytotoxic effect, albeit to a lesser extent than that observed with the combination of etoposide and Erlotinib, probably due to the low activity of the enzyme. Erlotinib, on the other hand, was not able to potentiate CPT’s effect, which might strengthen the notion that its activity modulates MDR. The different abilities of Erlotinib and Gefitinib should be further established, as they might require dose adjustments or different treatment durations.

The growing evidence pointing to the existence of tumor-initiating stem cells and their role in tumor resistance, progression, metastasis, and cancer relapse after anti-cancer treatments, underlines the great importance in understanding their biological activity and subsequent ability to respond to specific treatment protocols. Although we show here that the initial stages of tumor growth, represented by the formation of tumorspheres, might be involved in extreme changes of the activity and expression of essential enzymes due to complex cellular mechanisms, these effects should be further elucidated, as the mammosphere system raises more questions than answers.

## Conclusion

Understanding the process of early tumor development could be of great value, as cancer, if not cured, could become a controlled illness, reducing mortality rates and increasing life expectancy and quality for patients. Indeed, in this study we show that the understanding of the biological activity of essential cellular enzymes, such as topoisomerases, at early cancer development stages, may suggest treatment strategies for the development of potent, more effective treatment protocols and that the combination of TKIs and topoisomerases inhibitors could be effective against breast cancer.

## Electronic supplementary material

Additional file 1: ***Mammosphere*** **-** ***derived cells retain their ability to generate spheres for several passages.*** MCF7 cells were cultured as single cells on non-adherent plates, at a density of 20,000 cells\ml, in the presence or absence of fetal bovine serum, to form sphere-like structures (mammospheres). Cells grown without serum were cultured in DMEM:F12 medium solution mix, supplemented with 0.4% bovine serum albumin (BSA), 20 ng/ml EGF (Sigma-Aldrich, Israel), 10 ng/ml bFGF (Beit HaEmek Biological Industries, Israel), and 5 μg/ml insulin (Sigma-Aldrich, Israel). Mammospheres were collected after 7–10 days in culture, enzymatically and mechanically dissociated and resuspended as single cells to form the next generation of mammospheres, in order to evaluate stem-like self-renewal ability. (TIFF 777 KB)

## Abbreviations

3AB: 3-Aminobenzamide
bFGF: Basic fibroblast growth factor
CPT: Camptothecin
CSC: Cancer stem cell
EGF: Epidermal growth factor
FCS: Fetal calf serum
GFs: Growth factors
PARP: Poly-ADP-Ribose Polymerase
TK: Tyrosine kinase
TKI: Tyrosine kinase inhibitor
Topo: Topoisomerase.
