# Supplementary figures and images for: Modification of topoisomerases in mammospheres derived from breast cancer cell line: clinical implications for combined treatments with tyrosine kinase inhibitors
Source: BMC Cancer. 2014 Dec 3;14:910. doi: 10.1186/1471-2407-14-910 (PMC4289278; doi:10.1186/1471-2407-14-910)

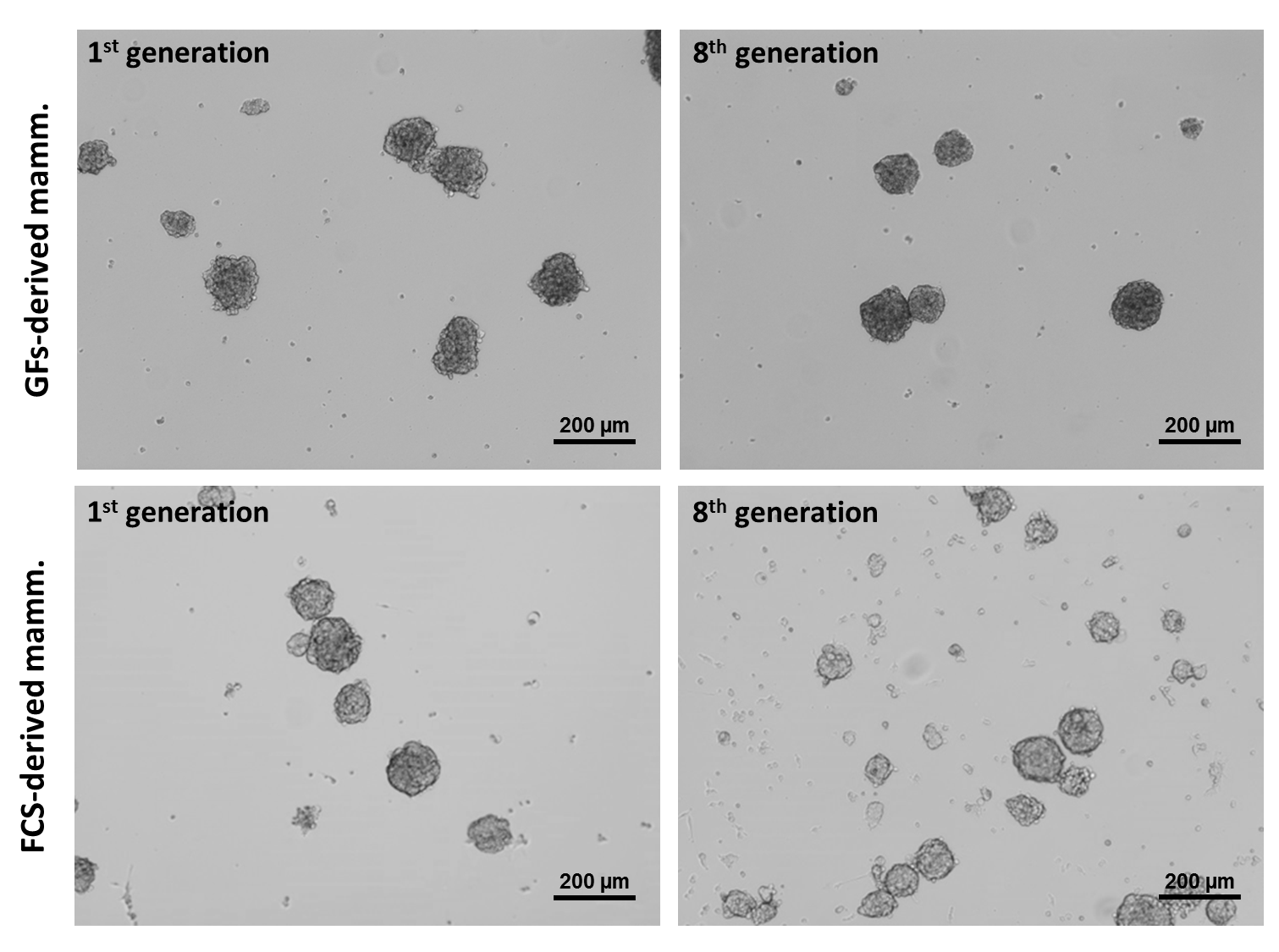

Supplement: Supplementary file 1 — Additional file 1: Mammosphere - derived cells retain their ability to generate spheres for several passages. MCF7 cells were cultured as single cells on non-adherent plates, at a density of 20,000 cells\ml, in the presence or absence of fetal bovine serum, to form sphere-like structures (mammospheres). Cells grown without serum were cultured in DMEM:F12 medium solution mix, supplemented with 0.4% bovine serum albumin (BSA), 20 ng/ml EGF (Sigma-Aldrich, Israel), 10 ng/ml bFGF (Beit HaEmek Biological Industries, Israel), and 5 μg/ml insulin (Sigma-Aldrich, Israel). Mammospheres were collected after 7–10 days in culture, enzymatically and mechanically dissociated and resuspended as single cells to form the next generation of mammospheres, in order to evaluate stem-like self-renewal ability. (TIFF 777 KB) [file 12885_2014_5139_MOESM1_ESM.tiff]
